# Food-Grade Bacteria Combat Pathogens by Blocking AHL-Mediated Quorum Sensing and Biofilm Formation

**DOI:** 10.3390/foods12010090

**Published:** 2022-12-24

**Authors:** Kirsi Savijoki, Paola San-Martin-Galindo, Katriina Pitkänen, Minnamari Edelmann, Annika Sillanpää, Cim van der Velde, Ilkka Miettinen, Jayendra Z. Patel, Jari Yli-Kauhaluoma, Mataleena Parikka, Adyary Fallarero, Pekka Varmanen

**Affiliations:** 1 Department of Food and Nutrition, University of Helsinki, FI-00014 Helsinki, Finland; 2 Drug Research Program, Division of Pharmaceutical Biosciences, University of Helsinki, FI-00014 Helsinki, Finland; 3 Faculty of Medicine and Health Technology, University of Tampere, FI-33014 Tampere, Finland; 4 Drug Research Program, Division of Pharmaceutical Chemistry and Technology, University of Helsinki, FI-00014 Helsinki, Finland; 5 Fisher Scientific Ltd., FI-01620 Vantaa, Finland

**Keywords:** *Chromobacterium violaceum*, violacein, AHL, quorum-sensing, anti-biofilm, food, lactobacilli, *Propionibacterium*, propionibacteria, acetic acid, propionic acid

## Abstract

Disrupting bacterial quorum sensing (QS) signaling is a promising strategy to combat pathogenic biofilms without the development of antibiotic resistance. Here, we report that food-associated bacteria can interfere with the biofilm formation of a Gram-negative pathogenic bacterium by targeting its AHL (acyl-homoserine lactone) QS system. This was demonstrated by screening metabolic end-products of different lactobacilli and propionibacteria using Gram-negative and biofilm-forming *Chromobacterium violaceum* as the QS reporter and our anti-QS microscale screening platform with necessary modifications. The method was optimized in terms of the inoculation technique and the concentrations of D-glucose and L-tryptophan, two key factors controlling the synthesis of violacein, a purple pigment indicating the activation of the QS system in *C. violaceum*. These improvements resulted in ca. 16-times higher violacein yields and enabled revealing anti-QS effects of *Lactobacillus acidophilus*, *Lentilactobacillus kefiri*, *Lacticaseibacillus rhamnosus* and *Propionibacterium freudenreichii*, including new cheese-associated strains. Our findings also suggest that acetate and propionate excreted by these species are the main factors that interrupt the QS-mediated signaling and subsequent biofilm growth without affecting the cell viability of the *C. violaceum* reporter. Thus, the present study reports a revised anti-QS screening method to accurately define new bacteria with an ability to combat pathogens in a safe and sustainable way.

## 1. Introduction

Quorum signaling (QS) is a central mechanism used by bacteria to coordinate biofilm formation and pathogenesis [1,2,3]. Activation of this cell-to-cell communication system depends on the cell density and involves the production, release and detection of extracellular signaling (autoinducing—AI) molecules, which in Gram-negative bacteria, are passively diffusing acylated homoserine lactones (AHLs) [4]. Some Gram-positive bacteria, mainly Firmicutes and Actinobacteria, are also equipped with AHL-based QS systems, possibly acquired by horizontal gene transfer from Gram-negative bacteria [5]. Since QS-mediated biofilm formation is closely linked with increased antibiotic tolerance and pathogenesis, discovering antagonists against the QS-controlled cellular pathways has attracted considerable interest, since agents that target QS place low selective pressure on the bacterial cell and, thus, may be used to safely and sustainably prevent the formation of antibiotic-resistant and -tolerant biofilms by pathogenic bacteria [6,7,8,9].

In addition to controlling biofilm formation and antibiotic tolerance, bacteria can also use QS signaling as a competing strategy against other microbes co-inhabiting the same environment [10,11,12,13]. Health-promoting i.a., probiotic, organisms have gained particular interest as an alternative option to antibiotics to combat pathogenic bacteria due to their ability to prevent pathogenic bacteria from forming biofilms without inducing strong selective pressure on the pathogen, thereby demonstrating an advantage over conventional antibiotics [14,15,16,17,18]. Gram-positive propionibacteria—notably, *Propionibacterium freudenreichii*—represent GRAS organisms with promising probiotic traits [19]. These bacteria belong to the phylym Actinobacteria that is known for comprising species with a unique ability to produce secondary metabolites with high therapeutic potential [20]. While propionibacteria are reported to exert beneficial anti-mold, anti-yeast, antibacterial and even anti-cancer effects, and in combination with probiotic lactobacilli to provide synergistic effects [20,21,22,23], their ability to interrupt QS signaling has not yet been systematically studied [24].

Gram-negative *Chromobacterium violaceum* has shown high promise to screen anti-QS activities that could be used to prevent the biofilm formation of Gram-negative bacterial pathogens harboring an AHL-mediated QS system [17,25,26,27,28,29,30,31,32,33,34,35]. Interruption of the QS signaling in Gram-negative bacteria can be achieved by (i) preventing the synthesis of AHL molecules or the expression of QS regulon genes by QS inhibitors (QSIs) or by (ii) quorum quenching (QQ) that occurs by inactivating the AHL molecules by hydrolytic enzymes [25,26]. In *C. violaceum*, QS-dependent biofilm formation is coordinated by CviI and CviR (homologs of the well-established key factors LuxI/LuxR), which also regulate the *vioABCDE* operon coding for a purple secreted violacein [25,36,37,38]. Our previously established miniaturized (96-well microtiter plate) screening format is based on using wild-type *C. violaceum* (ATCC31532) and its mutant derivative with a disrupted *cviI* gene (CV026) [39] as reporters. This method enabled the screening of QSI and QQ activities using reduced violacein to indicate compounds with an antibiofilm effect, and when combined with a parallel cell viability analysis, this method allows for indicating compounds having only a bactericidal effect [27].

In the present study, we sensitized our previously established anti-QS screening method to identify QSI and QQ activities from complex microbial samples. We simplified the inoculation technique and optimized the screening growth medium in terms of L-Trp and D-Glc concentrations to maximize the violacein production in *C. violaceum* [38,39,40]. We validated the revised method with previously identified flavonols possessing known QSI and QQ activity [27,28], then tested its performance on metabolic end-products derived from selected lactobacilli and propionibacteria, including newly isolated *P. freudenreichii* strains. Our study identifies new *Lactobacillus* species with anti-QS activity and, for the first time, propionibacteria with anti-QS activities against the biofilm-forming *C. violaceum*. We conclude that the revised method performs well on microbial samples and demonstrates the benefit of confirmatory/complementary reporter systems for identifying mechanistic details underlying pathogen exclusion.

## 2. Materials and Methods

### 2.1. Bacterial Strains and Culture Media

Details of the bacterial strains used are listed in Table 1. Lactobacilli were routinely grown at the indicated temperatures on commercial MRS agar (Becton Dickinson (BD), Franklin Lakes, NJ, USA) before propagation in modified MRS broth (mMRS) with the following: 10 g L^−1^ tryptone pancreatic digest of casein, 10 g L^−1^ beef extract powder, 5 g L^−1^ Bacto^TM^ yeast extract, 1 g L^−1^ Tween 80, 2 g L^−1^ ammonium citrate tribasic, 5 gL^−1^ sodium acetate, 0.1 g L^−1^ magnesium sulfate heptahydrate, 0.05 g L^−1^ manganese II sulfate monohydrate and 2 g L^−1^ dipotassium hydrogen phosphate trihydrate (pH 6.5–7) [41]. The mMRS was supplemented with 1.0% *w*/*v* D-glucose (D-Glc) to obtain mMRSG. Propionic acid bacteria were grown on yeast extract–lactate medium (YEL) [42] composed of 10 g tryptone (Sigma-Aldrich, St. Louis, MO, USA), 10 g yeast extract (BD), 16.7 g of 60% *w*/*w* DL-sodium lactate (Sigma-Aldrich, St. Louis, MO, USA), 2.5 g K_2_HPO_4_ and 0.005 g MnSO_4_ supplemented with 1.5% agar (Difco, Becton, Dickinson and Company, Sparks, MD, USA) for four days at 30 °C under an anaerobic atmosphere (Anaerocult, Merck, Darmstadt, Germany) to obtain colonies. For liquid cultures, colonies of propionibacteria were suspended in YEL, where the cultures were grown for 3 days at 30 °C under microaerophilic conditions.

The *C. violaceum* strains (ATCC31532 and CV026) were routinely propagated on Luria–Bertani agar (LBA; Fischer Scientific, Leicestershire, UK) at +27 °C for 16 h. LBA was supplemented with 100 µg mL^−1^ kanamycin when culturing the CV026 mutant strain. To optimize the violacein production, the following screening growth media were used: (i) OXOID^TM^ tryptic soya agar (TSA) and TS broth (TSB), (ii) LB broth with 0.1% *w*/*v* yeast extract (LBYE), (iii) 0.5% *w*/*v* peptone, 0.5% *w*/*v* D-glucose (D-Glc), 0.25% *w*/*v* yeast extract and 0.03% *w*/*v* L-Trp (PDYT), (iii) PYT supplemented with varying D-glucose concentrations (0.0–1.0%, *w*/*v*) and (iv) PDY with varying L-Trp concentrations (0.0–0.1%, *w*/*v*). The optimized growth medium used for anti-QS and -biofilm screening assays was termed mPDYT, which contained 0.5% *w*/*v* peptone, 0.1% *w*/*v* D-Glc, 0.25% *w*/*v* yeast extract and 0.05% *w*/*v* L-Trp.

### 2.2. Isolation of New Propionibacterium freudenreichii Strains from Cheese

Two *P. freudenreichii* strains were obtained as follows. Briefly, a *P. freudenreichii* strain denoted as AS2 was isolated from Liechtensteinian cheese (three-month-cured Liechtensteiner, packaging date 27 July 2020) produced from thermized milk, and a strain denoted as AS51 was isolated from French cheese (Comte Prestige, packaging date 21 September 2020) produced from unpasteurized milk. Strains were isolated by mixing 1 g of small cheese pieces with 4 mL peptone–lactate solution and incubating at room temperature for 2 h with frequent vortexing. Serially diluted samples were plated on YEL agar or LGA (lithium lactate–glycerol agar) [42] and incubated anaerobically (Anaerocult A, Merck KGaA, Darmstadt, Germany) at 30 °C for 6 days. Putative *Propionibacterium* strains were selected from the YEL plates based on the colony morphology and from LGA based on the color change due to acid production. Individual colonies were re-plated and subjected to Gram staining and catalase testing. Subsequently, the presence of the *bluB/cobT2* gene specific/selective to *P. freudenreichii* was tested on the isolated bacterial colonies by PCR (polymerase-chain-reaction) using *bluB*/*cobT2*-specific primers (5′-CGGATCCAGTGATGA G GCGCGTGATCCCGA-3′ and 5′-ACGGAAGCTTGCGCTGCGGGAGCGCTACT-3′) [54]. Briefly, PCR was prepared using Phusion Mastermix (ThermoFisher Scientific, Waltham, MA, USA) with 3% (*v*/*v*) DMSO (ThermoFisher Scientific, Waltham, MA, USA). Ethidium bromide (0.5 μg mL^−1^; Sigma-Aldrich)-stained 0.8% (*w*/*v*) agarose (BioRad) gels were visualized with an Alpha Imager HP imaging system (ProteinSimple Inc. Corp., San Jose, CA, USA).

### 2.3. Collecting Spent Culture Supernatants from Lactobacilli and Propionibacteria

Selected lactobacilli and propionibacteria (Table 1) were cultured on solid media (MRS for lactobacilli and YEL for propionibacteria) as described above. Then, a bacterial colony was suspended in triplicate in 10 mL mMRS with 1.0% D-Glc for lactobacilli or in 10 mL YEL for propionibacteria. Lactobacilli were propagated for 72 h at 30 °C (*L. curvatus* 453, *F. fructivorans* 1579, *L. kefiri* 3070) or 37 °C (*L. rhamnosus* GG, *L. buchneri*, *L. reuteri*, *L. mucosae*, *L. acidophilus*) and propionibacteria for 3 days at 30 °C under microaerophilic conditions. The cell-free supernatants were obtained by separating the cells using centrifugation (21,100× *g*, 1 min, +20 °C) and storing the collected supernatants at −80 °C until use. For each culture, the optical cell density (OD) at 600 nm at the time of sample withdrawal and pH of the cell-free supernatants were measured.

### 2.4. Screening Anti-QS and Bactericidal Activities Using C. violaceum Reporters

**Figure 1:**
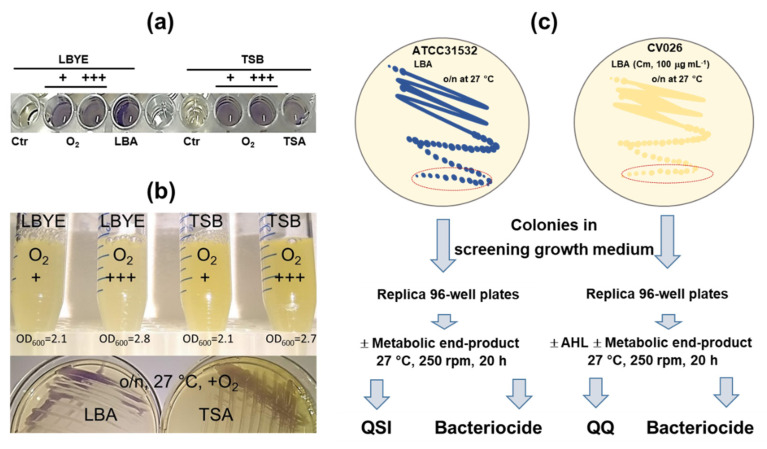
(**a**) Production of violacein in *C. violaceum* ATCC31532 cells growing in LBYE with (**b**) cell inoculates prepared from cells grown in liquid (LBYE or TSB) or solid (LBA or TSA) media. The first two were cultured under mild (+) or strong (+++) aeration and then suspended in LBYE at cell density OD_600_ = 0.001. (**c**) Workflow used for screening samples with QSI (ATCC31532), QQ (CV026) and bactericidal activities in the 96-well format. ATCC31532 and CV026 were first grown on LBA with/without chloramphenicol (Cm), then colonies were suspended in screening growth media with/without AHL (C6-HSL) under the conditions indicated. Pelleted violacein solubilized in ethanol was monitored at 595 nm to determine the viability of the reporter cells after resazurin staining with λ_ex/em_ = 560/590 nm.

In the optimized screening method, the reporter cells were grown on LBA (ATCC31532) or LBA/Cm (CV026) overnight at +27 °C, and cells were suspended in mPDYT (0.5% *w*/*v* peptone, 0.1% *w*/*v* D-Glc, 0.25% *w*/*v* yeast extract and 0.05% *w*/*v* L-Trp) to obtain OD_600_ = 0.01 with (CV026) or without (ATCC31532) 0.5 μM *N*-(β-ketocaproyl)-L-homoserine lactone (C_6_-HSL) (Cayman Islands; Figure 1a). Then, 200 μL of each cell suspension with/without the desired agents (flavanols F247 and 3896 with anti-QS activity, purchased from TimTec LLC, Tampa, FL, USA), controls (quercetin and azithromycin at 400 µM in DMSO as anti-QS and bactericidal controls, respectively) [27,28] or spent culture supernatants (in 5, 10, 15 and 20 µL aliquots) was added to wells in two parallel 96-well microtiter plates (Falcon^TM^ (tissue culture treated, polystyrene), BD). Then, the plates with/without the tested agents were shaken (250 rpm) overnight at 27 °C. The bacterial spent culture supernatants were tested in 5 and 10 µL aliquots using similar volumes of bacteria-free culture medium (YEL and mMRS supplemented with/without 1.0% D-Glc *w*/*v*) as the background controls. Meanwhile, 400 µM quercetin (in DMSO) and 400 µM azithromycin (in DMSO) were used as controls to monitor QSI and bactericidal activities [27]. 

Non-soluble violacein was obtained from the first 96-well plate by pelleting the cells and the violacein using centrifugation (3100× *g* for 20 min at 20 °C). Culture media was removed by pipetting using tips pre-wetted with ethanol to prevent violacein binding to the tips. The pelleted violacein was solubilized in 96% *v*/*v* ethanol (200 μL), supported by 96-pin microplate replicator (Boekel Scientific™ 140500) for better dispersal and mixing. Soluble violacein was separated from cell debris by centrifugation as described above, then transferred (150 μL) to new 96-well plates as above. The violacein yield was recorded using a PerkinElmer Victor3 reader (PerkinElmer, Waltham, MA, USA) at 595 nm. The bactericidal activity (viability) of the cells with/without the tested agents was assessed by adding resazurin (Sigma-Aldrich, St. Louis, MO, USA) [55,56] at a final concentration of 200 μM per well to the second 96-well plate. The plate was shaken (200 rpm) in the dark and, after 30 min, centrifuged (4000 rpm for 20 min at 20 °C) to remove the interfering violacein and cells. The recovered supernatants were transferred to a new plate and the fluorescence of the cell samples in each of the 96 wells was recorded at λ_ex_ = 560 nm, λ_em_ = 590 nm using the PerkinElmer Victor3 reader. Each experiment was repeated several times using two biological and three technical replicate samples.

### 2.5. HPLC Analyses of Spent Culture Supernatants

For D-Glc, selected key metabolites (succinate, lactate, acetate, propionate and ethanol) were quantitatively determined from the cell-free spent culture supernatants using an HPLC method previously described [57] but with the following modifications. Briefly, the cell-free spent culture supernatants were diluted with MilliQ water 1:5 (*v*/*v*) and syringe filtered (0.45 µm). Then, the filtered analytes were subjected to an HPLC system (Waters Alliance separation module e2695, Milford, MA, USA) equipped with a photodiode array detector (PDA; Waters 717) and a refractive index detector (RI; Waters 2414). The flow rate of the mobile phase for 10 mM H_2_SO_4_ was set at 0.5 mL/min and the compounds were separated using a Hi-Plex H column (300 × 6.5 mm; Agilent, CA, USA) maintained at 40 °C. An RI detector was used to detect and quantify glucose, acids and ethanol. Quantification was based on an external standard method using standard curves of 0.12–40 µg per analyte injection.

### 2.6. Statistical Analyses

Statistical parameters (Z’, S/N and S/B) [58,59] for each assay were calculated throughout the screening process to monitor the assay performance and confirm the high quality of the obtained results. In paired comparisons, an unpaired *t*-test with Welch′s correction was used (GraphPad Software, Prism, La Jolla, CA, USA, version 8.0), where *p* < 0.05 was considered statistically significant and *p* < 0.001 highly statistically significant. cLog *p*-values of F267 and 2896 were calculated with ChemDraw Professional 18.2.0.48 software (PerkinElmer, Waltham, MA, USA).

## 3. Results

### 3.1. Reporter Grown on LBA Enhances Violacein Production in LBYE

In this study, the original *C. violaceum*-based miniaturized anti-QS screening [27] method was sensitized to perform better on complex bacterial metabolic end-products. Our earlier method was based on using a cell inoculum prepared from cells cultured overnight in TSB, which were refreshed/diluted in the same broth, cultured to the early logarithmic stage and then diluted in LBYE for screening purposes [27]. Bacterial cells are known to induce mutations during the stationary phase of growth [60]. Hence, to prevent the possible overgrowth of spontaneously arising mutants and increase the consistency/repeatability of the screening process, we simplified the first step of the method by preparing the cell inoculum directly from fresh colonies grown on a solid medium. Figure 1a compares the effects of different cell-inoculum types on the violacein production in ATCC31532 in the 96-well format. A visual inspection of ATCC31532 cells grown in LBYE inoculated with cells grown on LBA enabled us to determine those with the most intense violacein production, as evidenced by the appearance of blue pigment. We also monitored the violacein production in LBYE using cells grown in TSB and LBYE with strong (+++) or mild (+) agitation but did not find that it had an apparent effect on violacein production in LBYE.

One notable finding was the appearance of ATCC31532 colonies during growth, which were blue on LBA and colorless on TSA (Figure 1b), implying that QS-induced violacein production was already activated in cells growing on LBA. Both TS- and LB-based media contain tryptone that is rich in L-Trp, and LB also has a yeast extract that has been shown to support efficient production of L-Trp—an essential substrate for violacein synthesis [27,61,62]. Hence, we suggest that the presence of produced L-Trp is the most likely explanation for the formation of violacein in cells growing on LBA. Since inoculation of the screening growth medium with fresh *C. violaceum* ATCC31532 cells grown on LBA provided the highest violacein yield in the 96-well format, we decided to use this inoculation technique in the following optimization steps.

### 3.2. Violacein Production Is More Efficient in ATCC31532 Grown in PDYT Than LBYE

**Figure 2:**
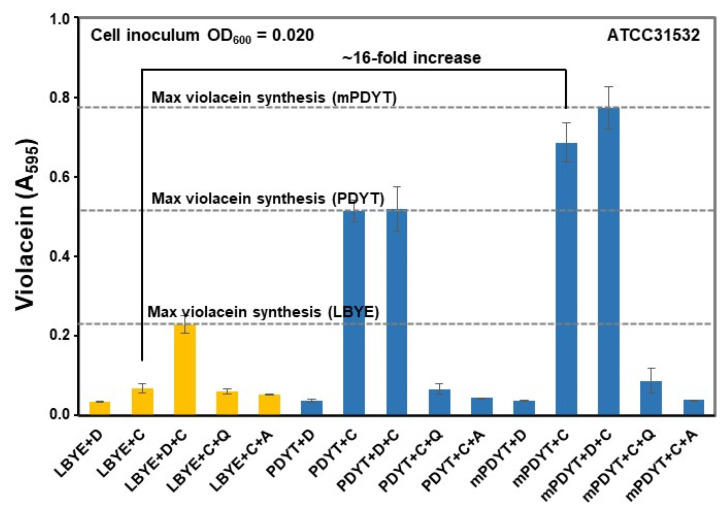
Violacein production of ATCC31532 in LBYE, PDYT and mPDYT in 96-well plates with/without 2% (*v*/*v*) DMSO. Each growth medium was inoculated with colonies grown on LBA at initial cell densities OD_600_ = 0.02. Violacein production was monitored at 595 nm. D, DMSO; LBYE/PDYT/mPDYT+D, growth media with DMSO (D) or *C. violaceum* cells (C). Azithromycin (A) and quercetin (Q) represent bactericidal and anti-QS controls, respectively, which were used at a 400 µM concentration. Error bars ± SD (*n* = 3–4). Grid lines, maximal violacein yields under the indicated conditions.

Since L-Trp could affect the violacein synthesis in *C. violaceum*, we next compared the violacein yields in growth media differing in terms of this amino acid. We tested a growth medium developed for another *C. violaceum* strain (denoted here as PDYT), containing more yeast extract than LBYE (0.25% *w*/*v*) and supplemented it with 0.03% *w*/*v* L-Trp [39]. We used the workflow outlined in Figure 1c to compare the violacein yields in LBYE and PDYT with/without 2% DMSO, a solvent routinely used when dissolving chemical compounds for anti-QS screening assays [27]. Figure 2 indicates that ATCC31532 cells secreted around 10-times more violacein in PDYT than in LBYE after overnight shaking at 27 °C. Thus, when PDYT provides more L-Trp or supports more efficient L-Trp production, this increases the violacein production efficiency in this QS reporter.

### 3.3. High D-Glc Inhibits and High L-Trp Stimulates Violacein Synthesis in C. violaceum

As PDYT provided higher violacein yields than LBYE with ATCC31532 as the reporter, we used this medium as the starting point to assess whether the violacein production efficiency could be further increased. PDYT contains D-Glc as the carbon source [39], which at high concentrations, has been shown to inhibit violacein synthesis [63,64]. Given that, we prepared a growth medium lacking D-Glc (PYT) and supplemented this medium with varying concentrations of D-Glc (0–1.0% *w*/*v*). Figure S1a shows the violacein production of ATCC31532 in PYT in response to different concentrations of D-Glc, as monitored in the 96-well format. Unexpectedly, the most abundant violacein yields were detected for cells grown without D-Glc, indicating that the addition of this carbon source impairs the induction of QS in ATCC31532. However, we could not quantitate the violacein yields reliably/consistently from these cultures. Furthermore, insoluble violacein appeared as floating pellicles, complicating its isolation by centrifugation. Since floating violacein was not seen with cell cultures grown on higher D-Glc concentrations and that could be easily isolated by centrifugation, we suggest that a higher cell mass aided pelleting the insoluble violacein. For this reason, we used the second-best concentration of D-Glc (0.1% *w*/*v*) in the subsequent optimization steps. Further, also worth noting is that separating cell-free culture supernatants from pelleted violacein was technically challenging because the pigment tended to bind and remain bound to pipette tips. To overcome this, we added an extra step to the protocol, which involved flushing the tips in 96% *v*/*v* ethanol before removing/transferring the culture supernatants.

PDYT contains 0.03% (*w*/*v*) L-Trp to maintain optimal violacein synthesis in *C. violaceum* [39]. Here, we tested if culturing *C. violaceum* with varying L-Trp concentrations affected the violacein production efficiency. For this purpose, both ATCC31532 and the CV026 mutant cells were cultured on PDY containing 0.1% (*w*/*v*) D-Glc, supplemented with L-Trp at different concentrations (0–0.1% *w*/*v*). Figure S1b shows that violacein synthesis was significantly more efficient with 0.07% L-Trp in ATCC31532 and 0.05% L-Trp in CV026 compared to 0.03% L-Trp in both strains. These findings indicate that the maximum violacein production in *C. violaceum* ATCC31532 and CV026 can be achieved using modified PDYT (hereafter mPDYT) consisting of 0.1% (*w*/*v*) D-Glc and 0.06% (*w*/*v*) L-Trp, with the combination of those concentrations supporting optimum violacein production by wild-type and mutant reporter cells.

### 3.4. Violacein Production in ATCC31532 Was Increased by >10-Times in mPDYT

Finally, the violacein production efficiencies in all tested growth media—LBYE, PDYT and mPDYT—were compared using ATCC31532 as the model. Figure 2 shows that violacein production in mPDYT was around 16-times more efficient compared to LBYE (without DMSO). An additional 13% increase in violacein yield was obtained when using modified PDYT (mPDYT) instead of PDYT. The assay performance values reflected by the screening window coefficient (Z’ = 0.5–1) and signal-to-background (S/B > 2) and signal-to-noise (S/N) ratios indicate that the screening assay performs well on the reporter strain (Figure S2a). The sensitized method was also validated using previously identified flavanols, 2896 (ST078866, TimTec) and F267 (ST079962, TimTec; Figure S2b), with reported anti-QS activities against ATCC31532 and its mutant derivative strain CV026, respectively [27,28]. Figure S2c compares the inhibitory profiles of the two flavanols with ATCC31532 and CV026 using quercetin and azithromycin as the known anti-QS and bactericidal controls [27], respectively. These comparative analyses indicate that both compounds at the 400 μM level inhibited QS in ATCC31532 and CV026 as efficiently as quercetin (with 2896) or with greater efficiency (with F267), which is in line with our earlier findings for these compounds [27,28]. The compound F267 was more effective against violacein production in CV026 than in ATCC31532, while 2896 was less effective in CV026 than in ATCC31532. Total violacein synthesis was inhibited by around 50% with 400 µM 2896 in CV026. Differences in the lipophilic properties (assessed by their cLog values) of these compounds could explain their differential QS-inhibitory profiles in ATCC31532 and CV026, as well as their possible mechanisms of action. F267 is likely to interfere with QS signaling outside of cells, whereas the more lipophilic 2896 could target CviR/CviR-AHL after entering cells. These findings indicate that the revised screening method fulfils the criteria for reliably identifying agents with anti-QS and bactericidal activity.

### 3.5. Lactobacilli Can Exert either QSI or QQ Activities against C. violaceum

**Figure 3:**
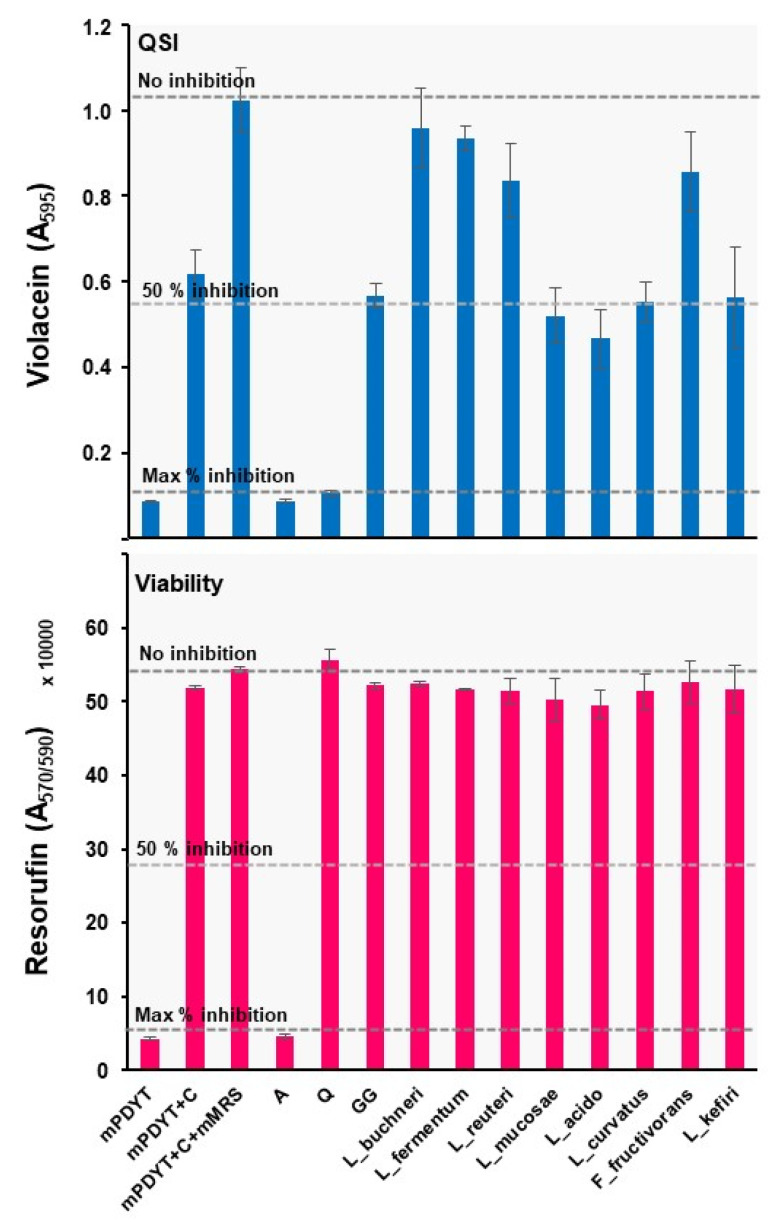
Anti-QS (blue) and bactericidal (pink) activities of the spent culture supernatants (10 µL) tested against ATCC31532. mPDYT, growth media; mPDYT+C, growth medium with ATCC31532; A, azithromycin; Q, quercetin. Error bars ± SD (n = 3–6). Z’ = 0.58; S/N = 7.20; S/B = 11.31. Grid lines, detected violacein levels with/without QS (Q) and bactericidal (A) inhibitors.

Lactobacilli are known to exert strong antimicrobial activity against pathogens, but only now are we beginning to appreciate the benefits of these activities in preventing disease [19,65,66,67]. Since secretion/excretion is likely to play a key role in combating pathogens, we tested spent culture supernatants withdrawn from the indicated lactobacilli grown on mMRS for anti-QS and bactericidal activities against *C. violaceum*. The workflow outlined in Figure 1b was used to prepare 96-well plates using ATCC31532 as the reporter, with optimized conditions for monitoring violacein production and cell viability with/without the metabolic end-product. Since mMRS is supplemented with 1.0% D-Glc (*w*/*v*) to maintain the physiological growth of lactobacilli, using this medium as the control with *C. violaceum* cells may hide the possible anti-QS effect due the presence of this carbon source in the medium. The final D-Glc levels in all spent culture supernatants were next measured to investigate this further, which in each case indicated that the tested lactobacilli had consumed nearly all of this carbon source, as the detected D-Glc levels with each culture supernatant were below 0.01% (*w*/*v*). All samples were then tested at volumes ranging from 5 to 20 µL, of which 10 µL (diluted 20-times in a screening assay) was the optimum volume enabling us to distinguish differences between test samples and controls. Control samples with mMRS at volumes above 10 µL inhibited violacein synthesis of ATCC31532 (data not shown). Figure 3 shows that the metabolic end-products secreted by *L. rhamnosus* GG, *L. mucosae, L. acidophilus*, *L. curvatus* and *L. kefiri* reduced violacein production by 50% or more since these species can block the induction of QS in ATCC31532.

**Figure 4:**
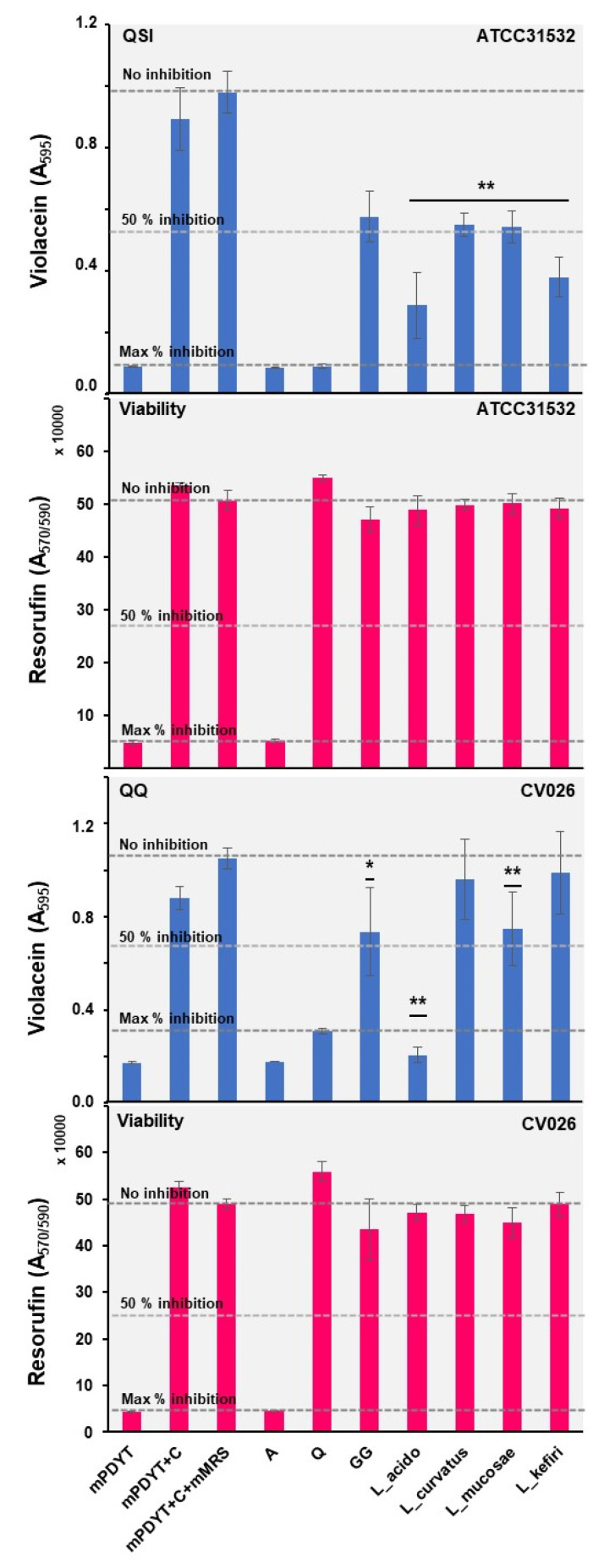
Anti-QS (blue) and bactericidal (pink) activities of the selected samples against ATCC31532 and CV026. mPDYT, growth media; mPDYT+C, growth medium with ATCC31532 or CV026; A, azithromycin; Q, quercetin. Error bars ± SD (*n* = 5–6). Changes in violacein synthesis in relation to mPDYT+C+mMRS; * *p* <0.05; ** *p* <0.001. Grid lines, detected violacein levels with/without QS (Q) and bactericidal (A) inhibitors.

Next, the most potent spent culture supernatants (GG, *L. acidophilus*, *L. curvatus*, *L. mucosase* and *L. kefiri*) were also tested against both ATCC31532 and CV026 to determine the possible QQ activity associated with these samples. Figure 4 shows the observed QSI, QQ bactericidal effects and indicates that each sample was able to reduce the total violacein yields in both ATCC31532 and CV026. Among the samples, *L. acidophilus* differed from the others in that the inhibition of violacein synthesis was equally efficient against both reporters, and that the anti-QS effect was more pronounced against CV026 than ATCC31532. Our findings are in line with earlier studies on *L. rhamnosus* GG and *L. acidophilus,* which indicated GG-producing anti-QS agents to combat Gram-negative *Escherichia coli* [68] and *L. acidophilus* interfering with the biofilm formation of the same pathogen by producing QQ activity [69]. Actinobacteria and Firmicutes (lactobacilli) are reported to produce AHL-degrading enzymes with an ability to prevent the biofilm formation of some Gram-negative bacteria [13,70,71]. These findings suggest that *L. acidophilus*, and possibly also *L. rhamnosus* and *L. mucosae*, may use such QQ enzymes to target AHL molecules already outside the reporter cells. It remains to be shown if the genes homologous to lactonases are present in the indicated three lactobacilli coding for enzymes that can hydrolyze the AHL molecules excreted by *C. violaceum*. In the case of *L. curvatus* and *L. kefiri*, our findings suggest that their metabolic products could interfere directly with the VioR-mediated functions within the cells, since no clear QQ activity could be detected with these samples against the CV026 mutant reporter. Only a minor anti-QS effect was detected with the metabolic products of *L. buchneri, L. fermentum*, *L. reuteri* and *F. fructivorans* against the wild-type ATCC31532 strain, which could be due to ineffective production of anti-QS agents under the tested conditions. Nevertheless, these species have shown promise in combating the QS systems of different Gram-positive bacteria [72,73].

### 3.6. Propionibacteria Interrupt QS Signaling in C. violaceum by QQ

**Figure 5:**
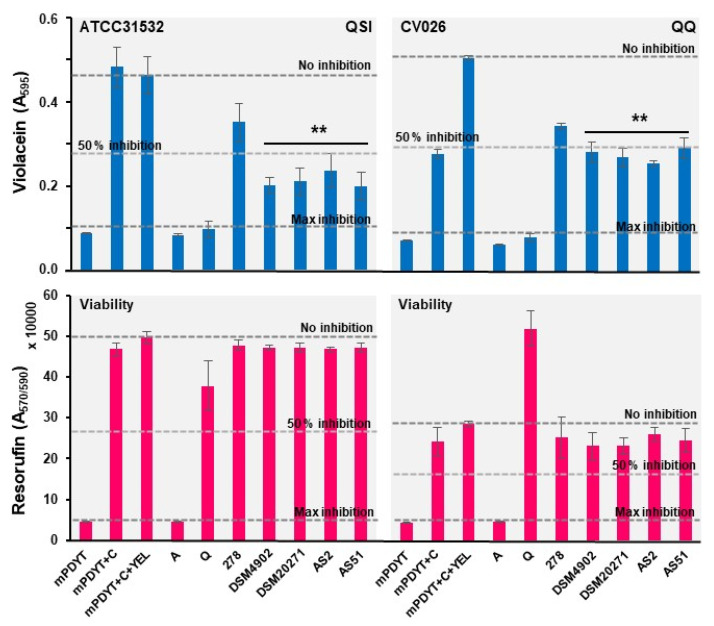
Anti-QS (blue) and bactericidal (pink) activities of the *Lactobacillus* and *Propionibacterium* cell-free supernatants against ATCC31532 and CV026. mPDYT, growth media; mPDYT+C, growth medium with ATCC31532 or CV026; A, azithromycin; Q, quercetin. Error bars, ± SD (*n* = 3–6). Changes in violacein synthesis in relation to mPDYT+C+YEL: **, *p* < 0.001. Grid lines, detected violacein levels with/without QS (Q) and bactericidal (A) inhibitors.

*P. freudenreichii* JS, DSM20271, B-3523 and B-4327 have been reported to have antibacterial effects against Gram-negative/-positive pathogens, either alone or in combination with a probiotic *Lactobacillus* [74]. Here, we tested whether two *P. freudenreichii*-type strains, a malted-barley-associated *Acidipropionibacterium virtanenii* (JS278) and two newly isolated *P. freudenreichii* cheese strains (AS2 and AS51), exerted anti-QS effects against *C. violaceum*. Figure 5 shows the QSI and QQ profiles detected after culturing the indicated reporters with 10 µL aliquots of metabolic end-products or YEL as the control. *Propionibacterium* samples were also tested in varying volumes, which indicated that volumes of the YEL control greater than 10 µL resulted in remarkably reduced violacein production (data not shown). The greatest inhibitory effect on violacein production (>50% compared to the YEL control cells) was obtained with *P. freudenreichii* samples, whereas secreted metabolic products of *A. virtanenii* resulted in only a 20–30% reduction in the violacein production of the ATCC31532 reporter. Since no obvious change in cell viability was observed with none of samples, we suggest that only the *P. freudenreichii* strains can combat the QS system of the reporter under the conditions tested. The inhibitory profiles for the same samples tested against the CV026 mutant reporter revealed that the anti-QS effect is restricted to ATCC31532, suggesting that the metabolite produced by *P. freudenreichii* strains must have entered the *C. violaceum* cells for blocking the VioI- and/or VioR-mediated functions. Hence, these findings suggest that *P. freudenreichii* can target a biofilm bacterium equipped with a conserved AHL synthase (VioI) and transcriptional regulator gene (VioR).

### 3.7. Acetic and Propionic Acids Can Block the Induction of QS of C. violaceum

**Figure 6:**
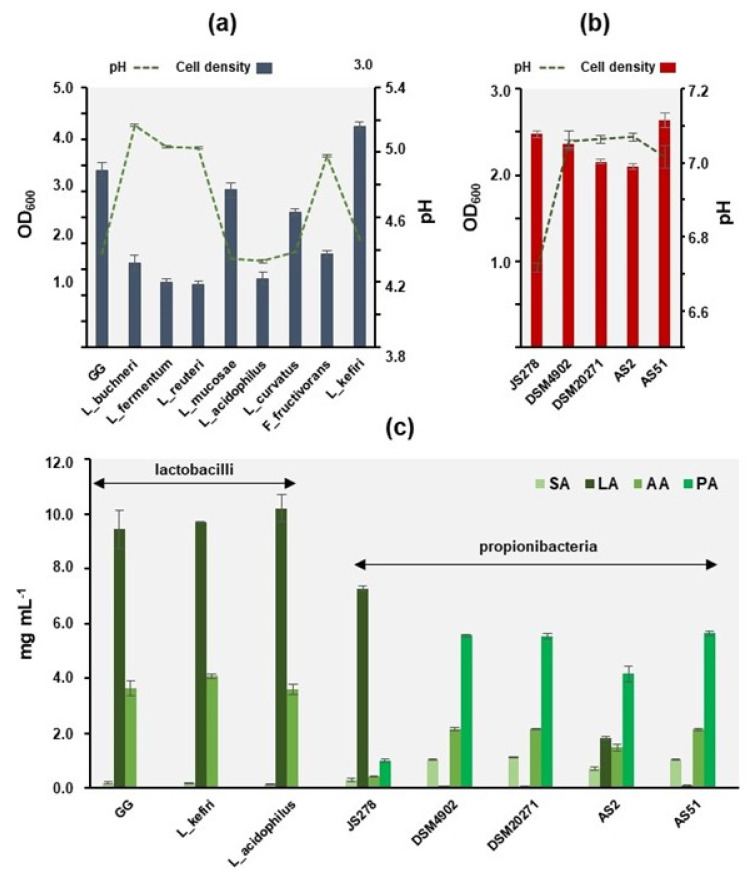
Cell density of the selected lactobacilli (**a**) and propionibacteria (**b**) at the timepoint of sample withdrawal and pH of the cell-free culture supernatants. (**c**) Concentration of key metabolic end-products secreted by the indicated lactobacilli and propionibacteria. SA, succinate; LA, lactate; AA, acetate; PA, propionate. Error bars ± SD (*n* = 4).

Organic acids that decrease the pH of the spent culture supernatants could provide another possible explanation for the reduced violacein production in *C. violaceum*. This assumption is based on a recent finding indicating that culture supernatants of other *L. rhamnosus, L. fermentum* and *L. acidophilus* strains induce pH-dependent inhibition of AHL-QS signaling in *P. aeruginosa* PAO1 [65]. A study on the *C. violaceum* strain MTCC8071 supported this idea by showing that violacein synthesis is pH-dependent; the optimum pH for violacein synthesis is 7.0, whereas at pH 6.0, the violacein synthesis is reduced by ca. 30% [40]. To investigate this further, we monitored the cell density of the *Lactobacillus* and *Propionibacterium* cultures and pHs of their cell-free supernatants. Figure 6a shows that *L. rhamnosus* GG-, *L. mucosae*-, *L. acidophilus*-, *L. curvatus* and *L. kefiri*-associated supernatants had the lowest final pH values (~4.2), while the highest (~5.0–5.2) were for those withdrawn from *L. buchneri*, *L. fermentum*, *L. reuteri* and *F. fructivorans* cultures. As the addition of cell-free supernatants reduced pH of mPDYT only slightly (e.g., GG, 6.9 ± 0.03; *L. acidophilus*, 6.8 ± 0.03; *L. kefiri*, pH = 6.9 ± 0.03) compared to the control sample with mMRS added to mPDYT (pH = 7.18 ± 0.01), we concluded that the detected anti-QS effect is mediated by factors other than pH. Instead, we suggest that the excretion/secretion of organic acids by the studied bacteria could have contributed to the detected anti-QS effects against the *C. violaceum* reporter. For example, lactic acid (LA), the major metabolic end-product of lactobacilli [75], has been shown to sensitize Gram-negative *E. coli* to antimicrobials by disrupting the outer membrane of the target cells [76] or by interrupting an AHL-mediated QS system [77]. Studies on propionic acid (PA), the main metabolic end-product of propionibacteria [19], have demonstrated that this short-chain fatty acid can be used alone or in combination with different organic acids or antibiotics to potentiate antibacterial or anti-biofilm effects [78,79,80,81]. Succinic acid (SA), a key intermediate metabolite of the tricarboxylic acid cycle, is also reported with antibacterial effects, e.g., against *Cutibacterium acnes* or with an ability to increase the antibiotic efficacy against *P. aeruginosa* PAO1 and alone to induce biofilm dispersal [82,83,84,85,86]. Acetic acid (AA) is another metabolic end-product that has been shown to potentiate the antibacterial effects against Gram-negative *Campylobacter jejuni* in combination with certain organic acids [79]. This acid can also coordinate the QS-mediated production of bacteriocins in lactobacilli to combat food-borne pathogens [87].

**Figure 7:**
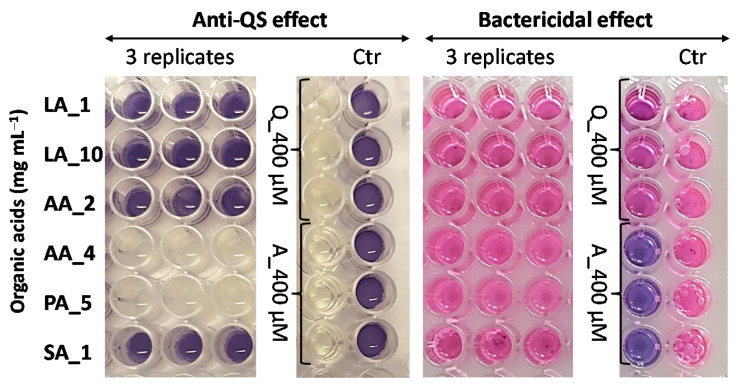
Anti-QS (blue versus colorless wells on the left) and bactericidal activity (pink versus blue wells on the right) of pure organic acids at the indicated concentrations (1–10 mg mL^−1^) against the *C. violaceum* ATCC31532 assessed in the 96-well format. LA, lactate; AA, acetate; PA, propionate and; SA, succinic acid. Quercetin (Q) and azithromycin (A) at 400 µM were used as anti-QS and bactericidal controls, respectively. Ctr, ATCC31532 cells with 10 µL of H_2_O were used as the positive control of an induced QS system and violacein production.

In view of these reports, we next assessed whether these organic acids (LA, PA, AA and SA) can interfere with the induction of QS in *C. violaceum*. To this end, we first subjected the supernatants to quantitative HPLC analysis to determine the concentrations of LA, PA, AA and SA among three selected *Lactobacillus*- and five *Propionibacterium*-associated supernatants (Figure 6a). The organic acid profile of the *A. virtanenii* cell-free supernatant differed from the propionibacteria in that we detected remarkably more LA (~7.5 mg mL^−1^) and less PA (~1.0 mg mL^−1^) compared to those determined from the *P. freudenreichii* samples. Next, we tested the anti-QS activity of these organic acids separately, at the concentrations determined for each in their respective cell-free supernatant, on the violacein production of wild-type *C. violaceum* ATCC31532. Figure 7 reveals that LA at 10 mg mL^−1^ did not affect the QS system of this reporter, which aligns well with the anti-QS screening results for control cells grown in the presence of YEL containing ~17 mg mL^−1^ LA (Figure 5). On the other hand, the anti-QS analyses with pure AA and PA at 4 and 5 mg mL^−1^ demonstrated that these acids could completely prevent the synthesis of violacein in ATCC31532 (Figure 7, colorless wells on the left), unlike the control with ATCC31532 cells grown in the presence of 10 µL H_2_O (Figure 7, blue wells on the left). Since 2 mg mL^−1^ AA did not affect violacein production, we suggest that the observed anti-QS effect associated with the *P. freudenreichii* samples was caused by PA. The bactericidal effect visualized by the reduction in resazurin (Figure 7, blue wells on the right) compared to resorufin (pink wells on the right) by metabolically active and viable cells confirms that both AA and PA at the indicated concentrations prevented violacein synthesis, since AA at 4 mg mL^−1^ decreased the cell viability by only 5% (SD = 0.05) and 5 mg mL^−1^ PA by 24 % (SD = 0.02). We also observed that the anti-QS effect associated with AA and PA was accompanied by a clear anti-biofilm effect; the reporter cells remained in a non-adherent/planktonic state, suggesting that AA at 4 mg mL^−1^ and PA at 5 mg mL^−1^ could be considered as genuine anti-QS agents. Since *P. freudenreichii* supernatants containing PA did not show a significant anti-QS effect against the mutant CV026 strain, we suggest that this acid interferes with AHL synthesis after penetrating cells to inhibit QS induction, demonstrated by the reduced violacein production in the wild-type ATCC31532 reporter. PA has long been used as a preservative in various food products due to its antioxidant effects and antimicrobial activity against yeast and bacteria, but the mechanisms underlying its effects are not fully understood. Taken together, the present study provides new information of how propionibacteria and lactobacilli fight against biofilm formation by linking a new mechanism of action to well-known organic acids, such as PA and AA.

## 4. Conclusions

The present study shows that the modified *C. violaceum*-based anti-QS screening method performs well on complex microbial samples, such as those involving spent culture supernatants. This was achieved by replacing the LBYE-based screening medium with a newly composed medium with optimized D-glucose and L-Trp concentrations (mPDYT), and using cells grown on LBA instead of in TSB for inoculating growth media for screening of the metabolic products. In such conditions, the maximum induction of violacein—the indicator of QS and biofilm formation in *C. violaceum*—was reached, as demonstrated by the up to 16-times greater violacein yields in mPDYT compared to those in LBYE. The increased dynamic range of the assay enabled us to link probiotic lactobacilli to new traits, such as *L. rhamnosus*, *L. acidophilus* and *L. mucosae,* with an ability to exert QQ activity to combat *C. violaceum* and presumably other Gram-negative bacteria equipped with similar AHL systems. We also demonstrated for the first time that *P. freudenreichii*, including two newly isolated strains, could use anti-QS activity to combat other bacteria. Further analyses demonstrated that the organic acids AA and PA, the central metabolic products excreted/secreted by the studied bacteria, were the most likely anti-QS factors to block violacein production and biofilm formation of *C. violaceum*. Taken together, the present study shows that the revised screening platform enables one to quickly (in less than three days) identify candidate bacteria with beneficial pathogen-exclusion traits.

## Figures and Tables

**Table 1:** Bacterial strains used to screen anti-QS and bactericidal activities.

Bacterial Species/Strain	Features and Traits	Origin
*Lacticaseibacillus rhamnosus* GG	Probiotic paradigm used as a dietary supplement and for the production of fermented foods; cultured at +37 °C	[43]
*Lactobacillus buchneri*	*Lactobacillus* used as an additive to improve the aerobic stability of silages; cultured at +37 °C	HAMBI69 ^(a)^
*Lactobacillus fermentum*	Probiotic *Lactobacillus* producing diverse and potent antimicrobial peptides; cultured at +37 °C	HAMBI73, [44]
*Limosilactobacillus reuteri*	Probiotic *Lactobacillus* used as a dietary supplement and for the production of fermented foods; cultured at +37 °C	HAMBI410, [45]
*Latilactobacillus curvatus*	Probiotic with excellent fermentation and health-promoting properties; cultured at + 30 °C	HAMBI453, [46]
*Limosilactobacillus mucosae*	Potential probiotic *Lactobacillus* encoding the cell-surface mucus-binding protein; cultured at +37 °C	HAMBI2674, [47]
*Lactobacillus acidophilus*	Probiotic *Lactobacillus* used as a dietary supplement and for the production of fermented foods; cultured at +37 °C	HAMBI80, [48]
*Fructilactobacillus fructivorans*	*Lactobacillus* used to produce fermented beverages. Can also act as a spoilage bacterium; cultured at +30 °C	HAMBI1579, [49]
*Lactobacillus kefiri*	Slime-forming probiotic *Lactobacillus* formed from kefir grains; cultured at +30 °C	HAMBI3070, [50]
*Acidipropionibacterium virtanenii* JS278	Propionic acid bacterium isolated from malted barley in Finland; cultured at +30 °C	[51]
*Propionibacterium freudenreichii*	Type strain; cultured at +30 °C	DSM4902, [52]
*Propionibacterium freudenreichii*	Type strain; cultured at +30 °C	DSM20271, [53]
*Propionibacterium freudenreichii* AS2	Propionic acid bacterium isolated from Liechtensteiner cheese (27.07.2020); cultured at +30 °C	This study
*Propionibacterium freudenreichii* AS51	Propionic acid bacterium isolated from Comte Prestige cheese (21.09.2020); cultured at +30 °C	This study
*Chromobacterium violaceum*	Biofilm-forming QS reporter strain; cultured at +27 °C	ATCC31532, [39]
*Chromobacterium violaceum* CV026	AHL-negative double miniTn5 disruption mutant of ATCC31532, km; cultured at +27 °C	NCTC13278, [39]

^(a)^ HAMBI Microbial Culture Collection, University of Helsinki, https://www.helsinki.fi/en/infrastructures/biodiversity-collections/infrastructures/microbial-domain-biological-resource-centre-hambi (accessed on 23 December 2022).

## Data Availability

The data is included in the article and Supplementary Materials.

## Supplementary Materials

The following supporting information can be downloaded at: https://www.mdpi.com/article/10.3390/foods12010090/s1, Table S1: Assay performance of the optimized screening method as defined by Z’, S/N and S/B values (*n* = 6); Figure S1: Comparison of violacein production in ATCC31532 and CV026 cultures prepared in PDY (with 0.1% *w*/*v* D-Glc supplemented with the indicated concentrations of L-Trp (%, *w*/*v*). The PDY cultures contained 2% *v*/*v* DMSO and were inoculated with ATCC31532 and CV026 cells at OD_600_ = 0.02. Error bars, ±SD (*n* = 3-4); Figure S2: (a) The assay performance of the optimized screening protocol as defined by the screening window coefficient (Z’), signal-to-background (S/B) and signal-to-noise ratios (S/N), *n* = 6. (b) Structures of quercetin, known QSI, and the previously identified flavonols, F267 and 2896 with known QSI/QQ activities. (c) Representative diaGrams of three experiments revealing anti-QS and bactericidal activities of F267 and 2896 against ATCC31532 and CV026 reporter strains using the optimized screening protocol. The compounds were tested at 100–40 µM (in DMSO). CD, cells with 2% DMSO; AI, 0.5 μM C6-HSL (Cayman Chemical, Ann Arbor, MI, USA); Q, Quercetin (400 µM, from 20 mM stock in DMSO); A, Azithromycin (400 µM, from 20 mM stock in DMSO). Violacein production was monitored using the Multiskan Sky microplate spectrophotometer and viability with a Varioskan LUX multimode plate reader (λex = 560 nm, λem = 590 nm). Error bars, ±SD (*n* = 3–4). Violacein production was monitored using the Multiskan Sky microplate spectrophotometer.
