# Supplementary material for: Food-Grade Bacteria Combat Pathogens by Blocking AHL-Mediated Quorum Sensing and Biofilm Formation
Source: Foods. 2022 Dec 24;12(1):90. doi: 10.3390/foods12010090 (PMC9818890; doi:10.3390/foods12010090)
Supplement: Supplementary file 1 [file foods-12-00090-s001.zip › Table S1.pdf]

Table S1. Assay performance of the optimized screening method as defined by Z', S/N and S/B values for six independent 96-microtiter plate analyses (n=6). Quercetin and azithromycin were used in each plate as the anti-QS and bactericidal controls, respectively.

Resorufin	i	ii	iii	iv	v	vi	n=6	Avg.	SD
Z'	0.77	0.72	0.83	0.77	0.82	0.79	Z'	0.78	0.04
S/N	15.42	13.07	19.96	15.42	19.42	19.73	S/N	17.17	2.91
S/B	8.42	6.27	8.79	8.42	8.03	6.07	S/B	7.67	1.19
**Violacein**	**i**	**ii**	**iii**	**iv**	**v**	**vi**	**n=6**	**Avg.**	**SD**
Z'	0.70	0.70	0.68	0.61	0.66	0.70	Z'	0.68	0.04
S/N	13.95	10.37	9.90	8.00	8.95	13.95	S/N	10.85	2.54
S/B	12.06	19.20	11.59	15.16	17.67	12.06	S/B	14.63	3.25
