# Supplementary figures and images for: Food-Grade Bacteria Combat Pathogens by Blocking AHL-Mediated Quorum Sensing and Biofilm Formation
Source: Foods. 2022 Dec 24;12(1):90. doi: 10.3390/foods12010090 (PMC9818890; doi:10.3390/foods12010090)

Figure S1

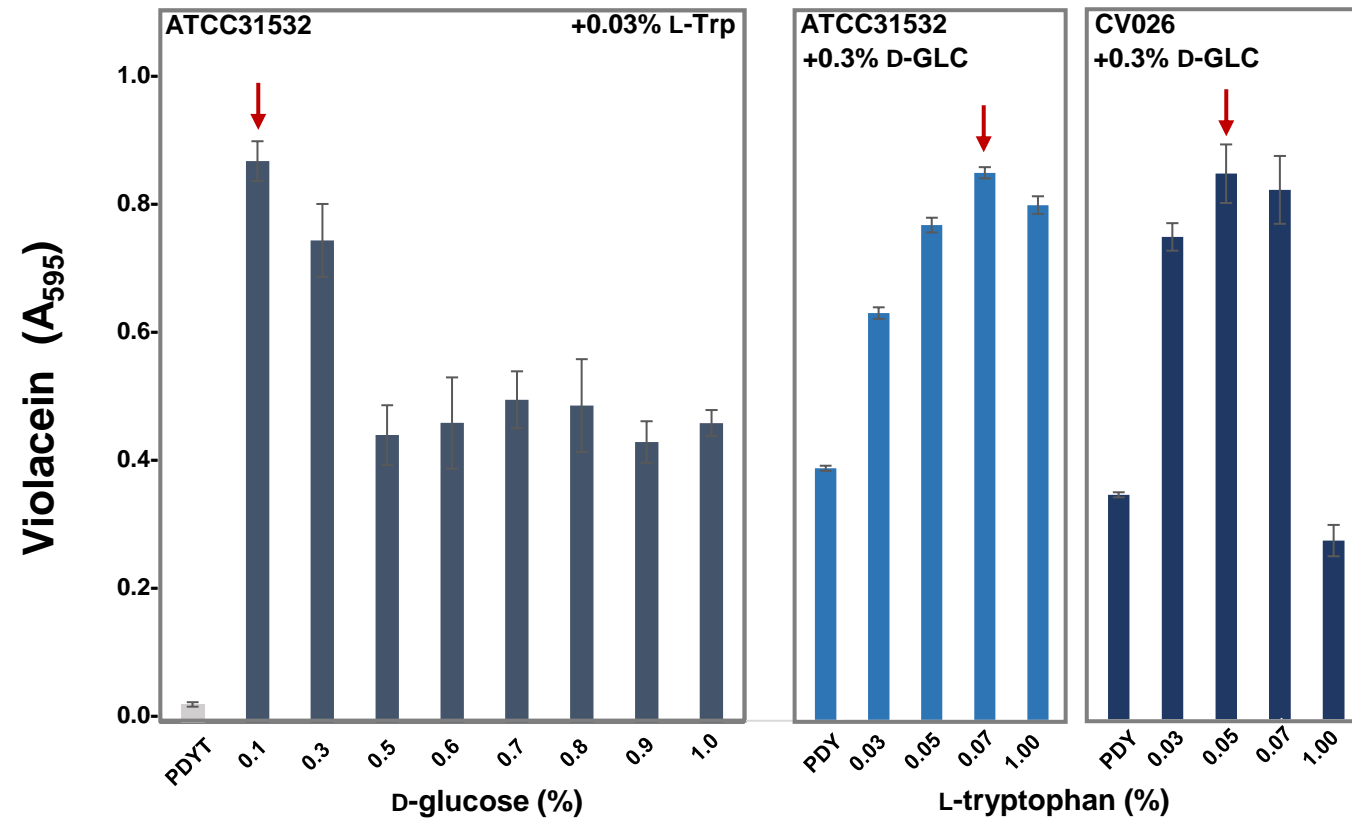

Supplement: Supplementary file 1 [file foods-12-00090-s001.zip › Figure S1.pdf]

Figure S2

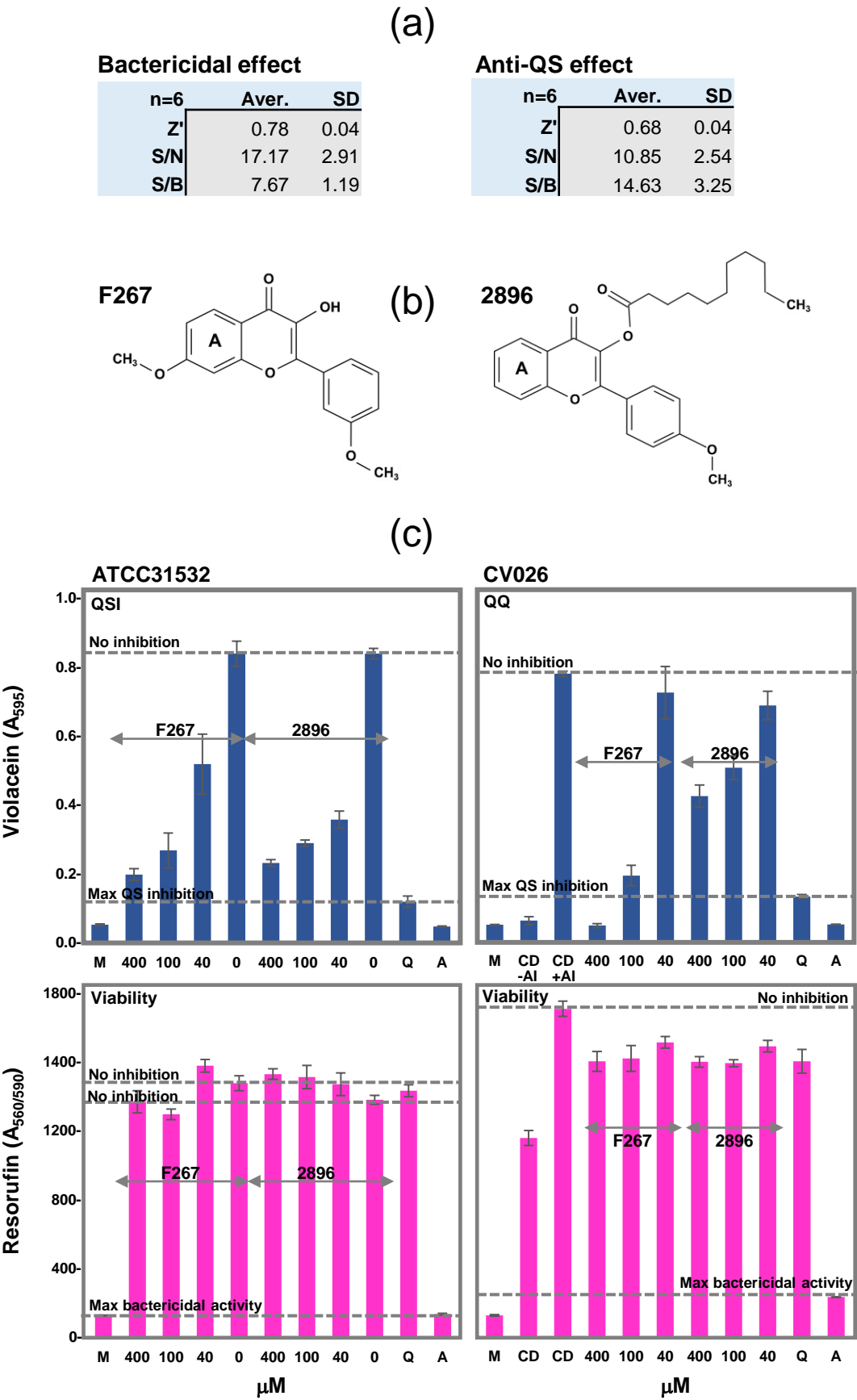

Supplement: Supplementary file 1 [file foods-12-00090-s001.zip › Figure S2.pdf]
